# Surgical Cases

**Published:** 1893-01

**Authors:** Edward Mahoney

**Affiliations:** Liverpool, England


### SURGICAL CASES.

EDWARD MAHONY, M. D., LIVERPOOL, ENGLAND.

Miss B., æt. thirty-six, consulted me first June 29th, 1889, with the following symptoms:

*Head:* Objects go round; feels as if she would fall forward; weekly pains; throbbing for years.

*Chest:* For two years wakes up feeling suffocated, not knowing where she is; worse before and during menses, which are every three weeks and scanty, and followed by thick, yellowish leucorrhœa. Chest attacks make her feel as if she would lose her reason.

*Mouth:* Bad taste mornings, with coated tongue.

*Stomach:* Pain at epigastrium, affecting breathing as though something were tight and she were breathing through something thick; can't either lie or sit down, walks about; aggravation often from 12 P. M. to 1 A. M.; goes through to back.

*Sleep:* Drowsiness 3 to 4 P. M.

*Fever:* Perspires easily with exercise, also in sleep, all over.

*General:* Worse in hot, thundery weather.

*Tumor:* Twelve months ago burning sensation in right jaw, and a lump, treated allopathically; getting larger about a month. Dead aching, also burning in lump at present, and down the arm. Had Arum$^{200}$, one dose, five days ago. She now received Sulph.$^{200}$, one dose.

July 2d.—Aggravation of pain next day some hours in arm, none since. Yesterday and to-day return of an old symptom, namely, twitching in right eye. No perspiration in sleep last night. Tumor in jaw smaller and can be more defined by manipulation.

*Further information:* Weak back since childhood. Likes everything loose. Coffee, cocoa, and beer disagree. Menstrual pain is most in right ovarian region, and central pain goes through to back.

July 4th.—Ovarian pain is dull, acting hot, with feeling of swelling inwardly, and as though something might give way. To take at next period, Apis$^{200}$.

July 21st.—Feels much stronger, but face more swollen and substance very hard, with lump inside mouth immovable, opposite last tooth of same side, no pain, feeling of heavy weight at vertex, a little pain in face occasionally; at the period there was much pain the first day, relieved after five globules of Apis as directed. Sulph.$^{200}$, eight doses, one night and morning.

August 22d.—She sends the following notes:

July 22d.—Swelling on face larger, with constant aching pain, looking inflamed at night, also swelling on knee and very sore, with pain in hip and leg, especially in the groin and joints.

28th.—Swelling smaller on face and less pain.

30th.—A smarting, burning pain from shoulder to wrist and from hip to bottom of foot.

August 4th.—Pain continuing, causing a weakness in walking, with a drawing of the guides and muscles, also a creeping sensation, followed by a shiver.

11th.—Pain ceased, but suffering with my nerves; could not hold anything steady. I shook and trembled so, and could not bear the least noise; this lasted several hours, when the pain in the chest came on, and through to the back.

12th.—Pain continued from shoulder to wrist, and from hip to bottom of foot.

14th.—Monthly period with much pain in lower part of body lasting two days.

16th.—Former pains continuing. Swelling on the knee quite gone; pain under the arm and in the breast.

21st.—Pains much the same. No swellings. Swelling on the face a little smaller. I am feeling better in myself, and have had no headaches for more than a month. I still have a bad taste in my mouth in the morning that has been going on for the last month. The monthly period pain was worse and lasted longer. I get a little pain occasionally in the swelling on the face, the swelling inside is much the same as when I wrote before. It's not painful, but very hard. I get a good deal of pain down the side of my neck, but not actually in the swelling.

Regarding the above report as indicating a penetrating action of the Sulph. she received Sac-lac.

September 4th.—Still feels better in herself, swelling on face is decidedly smaller, and her color is certainly better. (Her complexion is dusky naturally.) Menses were much less painful than before.

October 4th.—Swelling on face nearly gone, only just a small substance scarcely noticeable; swelling on both knees gone; arm and breast still painful when using former, especially in a little enlargement between the collar bone and the breast (right), an aching sick feeling; this swelling has been getting larger for about a fortnight. No pain at all at period, but piles caused much suffering, which in measure continues. Health very much improved. Graph.$^{200}$, one dose.

October 7th.—I feel so much better in every way, the breast is rather more painful to-day, other things are better.

November 1st.—Pain in breast better, swelling much the same; the pain is a dull, sick feeling; swelling on face gradually decreasing; headache ever since taking a dose of Ena's fruit salt on the 17th ult. Nux-vom.$^{200}$, one dose.

November 28th.—Feeling very much better, indeed well except pain in bosom, and that on the whole is better; swelling on face nearly gone, and that on the breast is a little smaller.

December 27th.—Severe headache with sickness weekly; bad taste mornings; pain in bosom decidedly better. Sulph.$^{4000}$, in three powders, each containing eight doses, to be taken in water at each nisus.

January 13th, 1890.—Headaches, which are at occiput and vertex, less severe, and some hours shorter, accompanied by nausea and retching, a trembling all over, feels as if she must drop everything; bad taste, afternoon drowsiness and vertigo as at first; return of an old neuralgia, sudden in coming and going, right face and temple, worse from excitement or fright. Since a urinary stoppage ten years ago, relieved by catheter, has been subject at times to urging with inability to pass any urine, or very little, which is white. During the last month has had a drawing in hands and feet, especially right, after walking, with pains in middle of right hand and sole of foot. Nails of right hand sore and full. Had a number of warts on both hands, mostly right, eight years ago, which went away of themselves. Pil. Bell.$^{20}$, one every half-hour, when in pain.

January 23d.—Telegraph, whole of head and back very bad; taken eight doses without ease; face flushed (Med.?). In consideration of the tendency to neuralgia from fright, and the symptoms of extremities above mentioned, Lycopod.$^{200}$, was advised, and Lycop.$^{30}$ afterward sent.

February 7th.—Above headache lasted nearly three days and nights with nausea; retching was relieved, but pain increased until menses appeared. Pain still, but less. Hands and feet much better. S. L.

March 8th.—Feeling much better. Weekly headache, but not near so bad, can look closely at things without pain or pressure. Breast much the same, a good dead of pain sometimes. Creeping in hands and feet quite gone. Finger-nails very sore at times.

April 8th.—Feeling very well, but more pain in breast and under arm, with some swelling. Has had three bad headaches. More strain physically and morally.

May 16th.—Feeling very much better; head very well and less pain in breast until last few days, when there has been a most acute pain at right shoulder, going through to breast, like toothache, easier when keeping the arm perfectly quiet, worse putting the hand in cold water, or lifting or grasping anything tightly. Sepia$^{500}$, one dose.

29th.—Pain in shoulder no better. A good deal of pain also under arm near bosom, and very sore to pressure; no swelling or substance, relieved by bathing (hot?). A creeping drawing in shoulder when arm is still in the day. No headache; feels very well in herself; face more swollen the last few weeks. Amm-carb.$^{30}$, *nocti namque* until relief.

July 4th.—After three days, period appeared, and shoulder pain was relieved, but there is smarting still from back of shoulder through to breast. Menses came again in fourteen days, making her feel poorly. A day or two before, there came a small substance on the side of the neck, about the size of a nut, very hard, smarting the first day, but nearly all gone down again. Pain and pressure at vertex and down back, and she can't look close at anything; burning. Helonias$^{200}$, six doses.

August 30th.—Feeling much better, and head so much better. Some return, since last report, of several pains, but these are over now.

February 13th, 1891.—Return of symptoms; pain in lower back, causing difficulty in getting about; menses ceased one month. Calc-carb.$^{200}$, three doses.

March 6th.—Pain in back and stomach quite gone; menses returned; bad sick headache five days ago, leaving biliousness; substance in face larger and much pain at root of tongue, going through ear; swelling also at outside of throat, tender to pressure. Hep.$^{200}$, one dose.

April 9th.—Feeling very well in herself, face and throat still a little painful; much smarting pain in right breast, going through to shoulder, also under arm, much worse after sewing. Natr-mur.$^{200}$, one dose.

May 21st.—Felling very well. A little pain still as above.

June 5th.—Severe cough; much expectoration while dressing. Two sisters are ill with the influenza. Bry.$^{200}$ ter dii.

September 3d.—Return of odontalgia; worse after sewing, hot food, or drink. Natr-mur.$^{1000}$, one dose. *On examination*, nothing either in cheek or breast of swelling, hardness, or tumor of any sort.

September 8th.—Reports almost unbearable neuralgia of head and face, followed by exhaustion. Regarding this as medicinal, I sent S. L.

November 19th.—Feeling very well in herself, and has been for some time, no pain in bosom since neuralgia above reported, but weekly headaches, especially on vertex and in eyes, sometimes with vomiting, and itching rash on hands, burning after rubbing or scratching, and better from hot water; both forefingers constantly going stiff. Sulph.$^{200}$, weekly.

December 24th.—Great relief; no headache while taking weekly powders; bad sick headache on omitting them. Irritation on hands entirely gone, and general health very well. Sulph.$^{4000}$, weekly.

February 16th, 1892.—Still has headaches unless taking medicine, also on getting up from sitting there is pain at the bottom of the back; pains also on sitting, unless leaning forward, just the bottom of the bone; most after walking; several weeks. Sulph.$^{50m}$, weekly.

March 18th.—Return of pain in head the last month; cannot look at anything long. Pain still at bottom of back. Nitr-ac.$^{200}$, weekly.

April 4th.—Head better after an aggravation preceding the period; feels fairly well, but pain in back still. Nil.

### Remarks.

This patient came to me after having been under the care of an allopathic surgeon of celebrity, indeed, the operating surgeon of a fashionable inland town in this country. I wrote to my patient a few days ago for particulars of this gentleman's opinion and treatments, and her reply is as follows:

"Dear Sir:—Yours to hand this morning. Four years ago the swelling first came in my face. I then had some ointment of the chemist to rub it with night and morning, which I did for several weeks; finding it got no smaller and not feeling very well, I consulted Dr. ——, and he said then it was indigestion and weakness, and advised me to go away for a change, which I did and was much better; the swelling went down a little, but not entirely away. I told Dr. —— I had been rubbing it with ointment, and he advised me not to do so. I was then under him a fortnight. Twelve months afterward it became worse again, and I consulted Dr. —— at once, and he at the first consultation said it was a growth, and must be operated on at once as there was nothing else for me.

"I was then under him three weeks. Then a few weeks after my return from Liverpool. Dr. —— acknowledged that he was wrong and that it would pass off in time."

I may add here that the patient told me Dr. —— had said to her, if you can get that tumor removed by any internal treatment whatever, I will believe in it, whether it is Homœopathy or anything else. I endeavored to persuade her to go and show herself to him when the tumor had quite gone, but she could not summon courage, being a sensitive, nervous person. For myself, I should certainly have said, in the language of the pathology of the day, that it was a malignant growth, both from its history, firm structure, and adherency, and the way in which the breast on that side threatened growths gave me anxiety for some months.

Speaking in Hahnemann's truer lines the growth may have been sycotic, but I quite think any allopath whatever, giving his unbiassed opinion would have pronounced the tumor one only to be eradicated by the knife. Dr. ——'s second opinion, as recorded by my patient, being, after several week's homœopathic treatment, which, as the notes show, had altered both the size and appearance of the growth, I regard as complimentary rather than otherwise. The patient is still troubled with headaches, but no one now would suspect anything but what is functional in her complaints, and were she only living in Liverpool and regularly presenting herself, the probabilities are in favor of her having got rid of these by this time.

